# Book Review

**Published:** 1950-04

**Authors:** 


### Reviews of Books

**Stillbirths.** By Ian Sutherland, D.Phil., F.S.S. Pp. xii, 93. London: Oxford University Press (Geoffrey Cumberlege). 1949. Price 7s. 6d.—This little book deals with a subject which has until comparatively recently received but scant attention. The author sets out to draw attention to the potential wastage which occurs in the first year of life and continues to examine the several causes, medical and social, which may have a bearing on this problem. The facts, carefully collected and sifted, are set out with an unbiased opinion. The style is easy, the tables and figures clear, and the statistical approach should not be beyond the limits of the average reader. There follows a summary, full list of references and an adequate index. So small a volume could be read by all including the more serious-minded final-year student, though the book will appeal most to research workers in this field and to those who are in daily contact with the problem of stillbirths and neo-natal deaths.

**Materia Medica and Pharmacy.** By R. R. Bennett, B.Sc., F.R.I.C. Fifth Edition. Pp. xxviii, 276. London: H. K. Lewis & Co. Ltd. 1950. Price 16s.—This little book contains essentially a concise account of the "drugs, chemicals and preparations" of the *British Pharmacopoeia*, 1948. For each drug the chemical formula, source and official dose are given and the therapeutic use is outlined in a sentence or two. The book includes a short glossary of pharmaceutical terms, tables of weights and measures, of metric and imperial "equivalents", and of doses arranged alphabetically. A concisely and clearly written account of incompatibility forms an appendix. The book should be a very useful adjunct to the standard textbooks of pharmacology. Compared with the *B.P.* it has the advantage that a drug and its official preparations are mentioned together. One criticism may be ventured: the drugs are grouped according to their pharmacological action, but the grouping differs from that found in those textbooks of pharmacology which are commonly used by medical students. It may be suggested that for the sake of uniformity and easier reference the author employs a more customary method of grouping in a following edition.

**The Middlesex Hospital, 1745-1948.** By Hilary St. George Saunders. Pp. 100. Illustrated. London: Max Parish & Co. Ltd. 1950. Price 8s. 6d.—This short work which can be read in a night covers the story of a great teaching hospital over two hundred years. It will appeal chiefly to the layman, for whom it is obviously written, and to the ex-Middlesex man, who will find much that is nostalgic in the illustrations and in the well-written text. Every doctor will, however, find some interest in the book, since the story is one of the progress of medicine in a voluntary hospital. " The walls of a great institution are also built of living stones, the men and women who work there and whose corporate labour makes its reputation." The " men and women " include not only the medical and nursing staff, but a vast host of ancillary workers. No branch of hospital life and work has been forgotten from the surgeons and physicians to the kitchen staff. The nursing staff is given a justly prominent place in the picture. The layout of the book is attractive and easy to follow; the absence of an index is the only defect.

**Isotopic Tracers and Nuclear Radiations, with Applications to Biology and Medicine.** By W. E. SIRI. Pp. xiii, 653. Illustrated. New York: McGraw-Hill Book Company Inc. 1949. Price 106s. 6d.—This book is primarily intended for the physicist " to bridge the gap between those books intended solely for the nuclear physicist and those which merely describe the results of research in which radioactive isotopes and nuclear radiations were used ". There is a serious need for a really good work dealing with the general principles of the application of tracers to biological research. Dr. Siri's book is divided into three parts. The first covering almost half the book is concerned with the physics of isotopes and nuclear radiations—the properties of nuclei, gamma-rays, beta particles, " heavy " charged particles and neutrons—each illustrated with useful tables and graphs. Short chapters on fission and radioactivity conclude with a complete reproduction of the comprehensive Seaborg & Perlman table of isotopes. The second part on methods and instruments describes the estimation of stable and of radioactive isotopes, the theory and design of the mass spectrograph, of Geiger Muller counters and ionization chambers and the standardization of radioactive samples. The book unfortunately includes no reference to the most recent use of scintillation counting. A brief review of the theory of tracer methods, the principles of internal dosimetry, ashing techniques, and the safe handling of radioactive materials, will be useful to the biologist. The third part, entitled " Biological and Medical Applications of Isotopes," written by Ellsworth C. Dougherty, is mainly devoted to a really excellent bibliography. It gives nearly 1,800 references, classified under the element concerned. Dr. Siri and his collaborators are to be congratulated on producing a very useful work of reference. The book is well arranged and ease of reference is an outstanding quality: the index is perhaps the weakest feature of the book and little more useful than the table of contents at the beginning.

